# Event-Related Alpha-Band Power Changes During Self-reflection and Working Memory Tasks in Healthy Individuals

**DOI:** 10.3389/fnhum.2020.570279

**Published:** 2021-01-25

**Authors:** Takahiro Matsuoka, Takaki Shimode, Toshio Ota, Koji Matsuo

**Affiliations:** Department of Psychiatry, Faculty of Medicine, Saitama Medical University, Saitama, Japan

**Keywords:** attention, event-related synchronization/desynchronization, alpha-band, EEG, working memory, self-reflection

## Abstract

Dysfunctional attentional control is observed in patients with mental disorders. However, there is no established neurophysiological method to assess attention in such patients. We showed a discrepancy in alpha-band power in the tasks that evoked internal and external attention event-related alpha-band power changes in healthy subjects during self-reflection (SR) and working memory (WM) tasks in a preliminary study. In this study, we aimed at elucidating event-related alpha-band power changes in healthy subjects during the tasks, addressing the shortcomings of the previous study. Sixteen healthy volunteers were examined for the event-related power (ERpow) change during the tasks. The results demonstrated the discrepancy of alpha-band ERpow at 8, 10, and 12 Hz in the parieto-occipital area between the WM and SR tasks for a period between a target stimulus and a command stimulus, where a participant switched to internal attention from external attention according to the SR task and remained at external attention according to the WM task. The results suggest that alpha-band ERpow in this area is associated with the direction of attention in response to cognitive stimuli, indicating that the findings of ERpow during the two tasks would potentially aid in the clarification of the pathophysiology of the dysfunctional change in attention in patients with psychiatric disorders.

## Introduction

Dysfunction of attentional control is observed in patients with mental disorders, including patients with organic localized injury in the frontal lobe, and also in those with psychiatric disorders such as mood disorders, schizophrenia, and neurodevelopmental disorders (Posner et al., 2019). For instance, patients with attention deficit hyperactive disorder (ADHD) have the essential feature of a persistent pattern of inattention that interferes with functioning or development (American Psychiatric Association, 2013).

Dysfunction of attentional control in ADHD presents behaviorally as the wandering of the task, lacking persistence, having difficulty sustaining focus, and being disorganized. It is not due to defiance or the lack of comprehension (American Psychiatric Association, 2013). To date, the diagnosis of ADHD by such inattention is determined by a clinical interview. Biological markers to assess inattention are required; however, to the best of our knowledge, there is no established neurophysiological method to do this.

For the neurophysiological mechanism in ADHD, a hypothesis based on dysfunction in controlling Default Mode Network (DMN) has been proposed (Castellanos et al., 2008; Biskup et al., 2016; Janssen et al., 2017; Kernbach et al., 2018). The DMN is a neural network that consists of brain structures distributed around the cerebral midline area, such as the midsagittal region of the frontal lobe, posterior cingulate gyrus, and precuneus. The DMN is known to activate at rest and when the attentional direction changes to internal, such as during a self-reflection task (Fuentes-Claramonte et al., 2019; Magosso et al., 2019), while it suppresses when the attentional direction changes into external, such as during a working memory task (Gusnard et al., 2001). However, little is known of the association between attentional direction and neurophysiological dysfunction in areas relevant to the DMN component in patients with ADHD. Electroencephalogram (EEG) studies are helpful to clarify the association because of the high resolution in the time domain.

Recent studies have shown that patients with ADHD had a decreased amplitude and prolonged latency of P300 of event-related potentials (ERP; Kaiser et al., 2020), and a reduced amplitude of mismatch negativity (MMN; Cheng et al., 2016). In contrast, changes in alpha-band oscillatory activity were closely related to attentional control, and the alpha-band power changes were associated with DMN activity (Bowman et al., 2017). However, the relationship between the alpha-band power and DMN activity is still controversial; the alpha-band power and the DMN activity positively correlated in the eye-opening condition, although they did not correlate in the eye closing condition (Mo et al., 2013).

Recently, we conducted a preliminary study of event-related alpha-band power changes in healthy subjects during the self-reflection (SR) and working memory (WM) tasks (Shimode et al., 2019). A significant discrepancy in alpha-band power was detected when the two tasks were attentionally pointed in opposite directions. Such a discrepancy in alpha-band power in the tasks evoked internal and external attention and may reflect transient DMN activation. However, there were a couple of limitations in the preliminary study. First, the frequency resolution was not enough. Employing the Fast Fourier Transformation Analysis with a time window of 250 ms resulted in limited frequency resolution in every 4 Hz bin. The limited frequency resolution potentially contains partial contamination of the alpha-band power by theta-band power, particularly in the lower alpha-band in N100 of ERP. The contamination may cause a pseudo increase in theta-band power. Second, the Japanese words in the SR and WM tasks were easy for the Japanese participants. The easy tasks may cause insufficient attentional direction during the two tasks, resulting in a discrepancy in lower alpha-band power between the two tasks. To address these shortcomings in the current study, the frequency resolution was improved in every 2 Hz bin employing the pre-filtering method, and the words used in the tasks were in English, which is a second language in Japan.

In the present study, we aimed at elucidating event-related alpha-band power changes in healthy subjects during the modified SR and WM tasks. We hypothesized that the discrepancy in alpha-band power between the SR and WM tasks was visible compared to the preliminary study.

## Materials and Methods

### Subjects

Sixteen healthy subjects were studied. The subjects were recruited by advertisements and word-of-mouth communication in the community. All subjects were right-handed and the mean ± SD age was 27.4 ± 4.6 years. All subjects were male. Female subjects were not included in this study because ADHD is more frequent in males than in females in the general population, with a ratio of approximately 2:1 in children and 1.6:1 in adults (American Psychiatric Association, 2013). Subjects were required to have a university degree as a minimum education requirement. Proficiency in English in participants was confirmed to not be a problem for proceeding with the task in this study. No subjects had any psychiatric disorders including ADHD according to the Diagnostic and Statistical Manual of Mental Disorders, 5th edition (American Psychiatric Association, 2013) in a clinical interview by senior psychiatrists. No subjects took any medications or had any physical diseases which could potentially influence cognitive and brain function. Any subject with abnormal EEG findings suggesting epilepsy and other organic brain disorders was excluded from the study by a psychiatrist with the certification of EEG expertise by the Japanese Society of Clinical Neurophysiology. This study was approved by the Ethics Review Board, Saitama Medical University (No. 834). All experiments were performed following relevant guidelines and regulations. After the study was fully explained to each subject, we obtained written informed consent from all participants.

### Task Procedure

**Figure 1:**
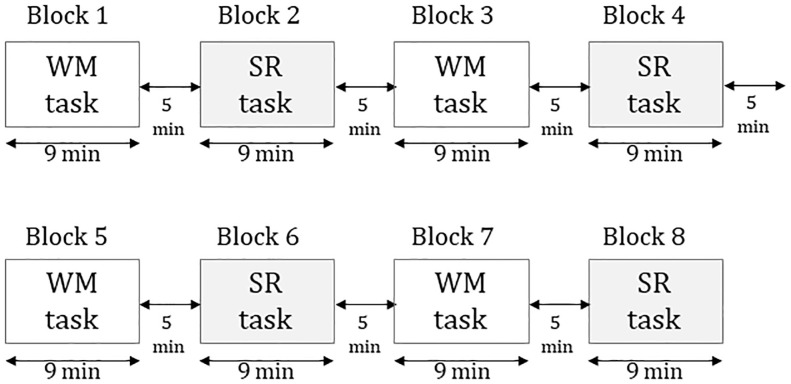
The procedure of the experiment consists of eight blocks in which four were working memory tasks and four were self-reflection tasks.

The task consisted of eight blocks for 110 min. Four blocks were the WM task, while the remaining four were the SR task. Each block included 56 trials for 9 min. The interval between blocks was 5 min (Figure 1). The total number of trials per subject was 224 for each of the WM and SR tasks. The WM and SR trials were displayed in the order. Subjects sat on a reclining seat in a relaxed manner. A white dot as a fixation point was displayed in the center of the black screen in a liquid crystal display monitor of 23 inches. The distance between the subject’s eye and fixation point was set at 150 cm.

#### Working Memory Task (WM)

The WM task was a modified Go-No Go task. The visual stimuli were six adjective words that were 5–8 “letters” in length in English. The words were presented for 50 ms in the center of the screen in a size of 6.5 cm × 18.5 cm in the WM task in a pseudo-random manner. The adjectives were “Brave,” “Careful,” “Strict,” “Flexible,” “Diligent,” and “Generous.” The words were chosen from a list of adjectives in a previous study (Aoki, 1971) so that the “desirability rate” was less than 4.5 degrees and the standard deviation was less than 1.

**Figure 2:**
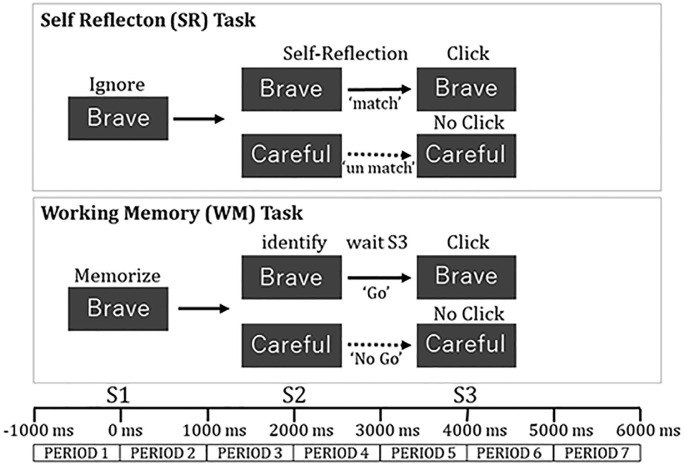
Task procedure in each trial for self-reflection task and working memory task.

A word as the warning stimulus (S1) was displayed. Subsequently, a word as the target stimulus (S2) was displayed 2 s after S1, and the command stimulus (S3) was presented 2 s after S2. The subjects were instructed to memorize S1, identify if a word of S2 was the same as a word of S1, and push a button using a computer mouse at S3. Subjects clicked the “Go” button at S3 when a word of S2 matched that of S1 (“Go” condition: 43% of trials) and did not click the button at S3 when no words of S2 and S1 matched (“No Go” condition: 57% of trials). A white dot was presented for 2 s after S3. The interval between the blocks was for 3 s The subjects were instructed to keep their eyes open during the words displayed and blink when the white dot was displayed (Figure 2).

#### Self-reflection Task (SR)

The SR task was presented in the same sequence as the WM. The subjects were instructed to ignore the meaning of a word at S1, then evaluate whether the meaning of the word at S2 was thought to match one of their personality traits, and click a “Go” button by computer mouse at S3 (“Match” condition). They did not click the button when the meaning of the word at S2 was not thought to match with one of their personality traits (“No Match” condition; Figure 2).

### EEG Recording

Multi-channel EEGs were recorded from 21 scalp sites (F3, Fz, F4, FC3, FCz, FC4, C3, Cz, C4, CP3, CPz, CP4, P3, Pz, P4, PO3, POz, PO4, O1, Oz, O2) using a digital EEG (Neurofax μ EEG-9100, NIHON KOHDEN Corporation). All scalp electrodes were digitally referenced to linked ears. Impedance in each electrode was less than 10 kΩ. The EEG signals were amplified by a bandpass of 1.6–120 Hz and sampled at a rate of 1,000 Hz. The onset of each stimulus and each response were marked digitally on a trigger channel.

### Data Analysis

#### Behavioral Performance

For the WM task, the mean reaction time was calculated in the “Go” condition. The rate of accurate response was calculated in the “Go” and “No Go” condition.

For the SR task, the mean reaction time in the “Match” condition was also calculated. As the accuracy could not be calculated, the discrepancy rate between “Match” and “No Match” conditions for the top three words frequently selected were used for the behavioral analyses. If the discrepancy rate was significant, the performance would be considered to be verified.

#### EEG

The EEG data during the trials in the “No GO” condition of the WM task and “No Match” conditions of the SR task was not used for the analysis, because these conditions were not properly monitored through the motor response. In the “GO” condition of the WM task, trials with an incorrect response and trials with a response time of more than 1,000 ms were excluded from the analysis. In the “Match” condition of the SR task, trials with a response time of more than 1,000 ms were also excluded from the analysis, because we considered that the response time meant that subjects inadequately performed the task in the epoch. Then, a total of 1,416 trials in the “GO” condition of the WM task and 1,538 trials in the “Match” condition of the SR task were selected for the analysis. Subsequently, a clinical neurophysiology instructor in the EEG section, certified by the Japanese Society of Clinical Neurophysiology, assessed the EEG data. The data of epoch with artifacts, including eye movement, muscle activity, electrocardiogram, and sweating were excluded from the analysis. Finally, 898 trials (63% of the total) in the WM task and 881 trials (58% of total) in the SR task were analyzed. The analysis was performed in the programming language under MATLAB 6.0 (MathWorks, Natick, MA, USA).

#### Event-Related Potential (ERP)

Event-related potentials in the “Go” and “Match” conditions were computed for both SR and WM tasks. Peak amplitude and latency in the negative and positive components after S1, S2, and S3 were also calculated for each task and each subject. Baseline correction was performed by subtracting the mean value of ERP between −1,000 and 0 ms from each time series of ERP.

#### Event-Related Power Change (ERpow)

The EEG data were exported into the computer for data analysis. The range of 1,000 ms before and 6,000 ms after the trigger signal of S1 was defined as one epoch. The epochs in the “Go” condition in the WM task and “Match” condition in the SR task were selected. These analyses were processed by the analytic program written in the language of SAS Software (SAS Institute, NC, USA). The digitally filtered time-series of 8 Hz, 10 Hz, and 12 Hz were extracted from the raw EEG epoch using Finite Impulse Response Filter for each area, each task, and each subject (Toma et al., 2002). In one epoch, variance values in the time-window of 250 ms were calculated: the first range of variance value in the time-window was −1,000 to −750 ms, the second one was −950 to −700 ms, and the series continued till the last one was 5,750 to 6,000 ms. The arithmetic mean of variance values was calculated using all trials for a task, a subject, an area, and a frequency-band. Finally, the time series of the power spectrum was obtained. To reduce the effect of inter-individual variability of absolute power, an event-related change in variance was estimated using the equation:

$$ER_{pow}{}_{{}_{xx}}\left(f\right)=\left(\log\left[P_{xx}\left(f\right)activation\right]-\log\left[P_{xx}\left(f\right)rest\right]\right)/SD_{xx}\left(f\right)$$

Pxx(f) activation: Power value in each time window, Pxx(f) rest: Mean power value in resting period (−1,000 to 0 ms), SDxx(f): Standard deviation of each time series of power values.

Color maps of spatial distributions of ERpow were drawn for PERIOD 5 and 6 during the SR and the WM tasks. We defined PO3 and PO4 as the regions of interest (ROIs) based on previous studies (Knyazev, 2012; Travis and Parim, 2017). The two studies demonstrated that the estimated cortical distribution of sources of scalp-recorded EEG activity using LORETA showed alpha-band activation in the parieto-occipital area during self-referential thoughts and transcendental meditation.

For each ROIs, an ERpow value for each time window was plotted on the midpoint of the time window; the first ERpow value for −1,000 to −750 ms was plotted on −875 ms, the second one from −950 to 700 ms was plotted on −825 ms, and the series continued till the last one for 5,750 to 6,000 ms was plotted on 5,875 ms. These analyses were processed by the analytic program written in the language of MATLAB 6.0 (MathWorks, MA, USA).

### Statistical Analyses

#### Performance Data

For the reaction time, a one-way analysis of variance (ANOVA) with the reaction time as a dependent variable and Task (SR and WM) as an independent variable was performed. For the discrepancy rate between “Match” and “No Match” conditions for the top three words in the SR task, one way ANOVA with the discrepancy rate as a dependent variable and condition (“Match” and “No Match”) as the independent variable was performed. To evaluate the habituation effect in the latter part of the experiment, a one-way repeated measures ANOVA with the reaction time as a dependent variable and the BLOCK (four blocks) as a repeated factor was performed for the SR and WM tasks. The degree of freedom was corrected using the epsilon value estimated through the Greenhouse-Geiser procedure. We used a statistical software of SAS version 8.0 (SAS Institute, Cary, NC, USA) with statistical significance set at *p* < 0.05.

#### EEG Data

For the ERP, the peak amplitude and latency in the negative and positive components after S1, S2, and S3 were obtained from each subject for both SR and WM tasks. Two-way ANOVA with the peak amplitude and latency in the negative and positive components as a dependent variable and with the task (two levels; WM and SR) and timing (three levels; after S1, after S2, and after S3) as an independent variable. With respect to ERpow, we used 28 samples per 7 s that did not overlap the time-window of ERpow in 140 data points per 7,000 ms. The PERIOD 1–7 were defined as follows: the baseline 0 ms was S1, PERIOD 1 was from −1,000 to 0 ms, PERIOD 2 was from 0 to 1,000 ms, PERIOD 3 was from 1,000 to 2,000 ms, PERIOD 4 was from 2,000 to 3,000 ms, PEROID 5 was from 3,000 to 4,000 ms, PERIOD 6 was from 4,000 to 5,000 ms, and PERIOD 7 was from 5,000 to 6,000 ms.

To confirm whether or not the selected ROIs showed significant ERpow increases in SR task, we used one-way ANOVA with ERpow as a dependent variable and with the condition (two levels; rest and activated) as an independent variable in each ROI for each part in PERIOD5 (earlier part: 3,000 to 3,500 ms, later part: 3,500 to 4,000 ms). PERIOD 1 was defined as the rest condition and PERIOD 5 was selected as the activated condition, because self-reflection was performed in PERIOD 5 during the SR task, resulting in opposite attentional direction between the SR and WM tasks.

For the time series of ERpow in ROIs, we used three-way repeated-measures ANOVA with ERpow as a dependent variable and with the task (two levels: WM and SR) and frequency (three levels: 8, 10, and 12 Hz) as independent variables and with PERIOD (seven levels) as a repeated factor. The degree of freedom was corrected using the epsilon value estimated through the Greenhouse-Geiser procedure. Scheffe’s multiple comparisons were employed as a *post-hoc* test to test the discrepancy in ERpow between WM and SR tasks.

## Results

### Performance Data

#### Reaction Time

The mean reaction time was 323 ± 114 ms in the “Go” condition in the WM task and 397 ± 171 ms in the “Match” condition in the SR task. The mean reaction time was significantly longer in the SR task compared to in the WM task (*F*_(1,30)_ = 5.0, *p = 0.03).

#### Accuracy in the WM task

Accuracy in the WM task was 97.6% in the “Go” condition and 99.6% in the “No Go” condition. Participants correctly performed the WM task with a rate of over 95%.

#### The Discrepancy Rate in the SR Task

All subjects clicked on a word in the “Match” condition in the SR task. The rate of the top three selected words was 38.9 ± 15.6% in the “Match” condition and 13.9 ± 12.1% in the “No Match” condition. The discrepancy of the two rates was statistically significant (*F*_(1,30)_ = 4.2, **p < 0.001), which meant that the task was properly performed in the subjects.

#### Habituation Effect

One-way repeated-measures ANOVA revealed that the main effect of the BLOCK was not significant in the SR task (*F*_(3,45)_ = 0.88, *p* = 0.42) and the WM task (*F*_(3,45)_ = 1.54, *p* = 0.24).

### ERP

**Figure 3:**
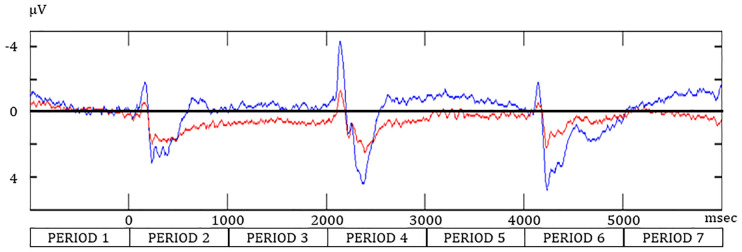
The event-related potential at Oz during the SR and WM tasks. Redline, the “Match” condition during the SR task; navy line, the “Go” condition in the WM task.

For the negative components, the peak amplitude was greatest after S2 in the WM and SR task (Table 1 and Figure 3). For the positive component, the peak latency after S2 was the longest in the WM and SR task. Two-way ANOVA revealed that task was significant for the amplitude in the negative component (*F*_(1,90)_ = 9.37, **p < 0.01), the latency in the negative component (*F*_(1,90)_ = 5.41, *p = 0.02), and the amplitude in the positive component (*F*_(1,90)_ = 15.33, **p < 0.01). Although task was not significant for the latency in the positive component (*F*_(1,90)_ = 0.04, *p* = 0.84), timing was significant (*F*_(2,90)_ = 23.21, *p < 0.01), although timing was not significant in others. No significant interaction between task and timing was observed.

**Table 1:** Peak latency and amplitude of N100, and P300 in Oz.

WM task	Post S1	Post S2	Post S3
N100			
Amplitude (μV)	−1.77	−4.29	−1.78
Latency (msec）	174	144	145
P300			
Amplitude (μV)	3.17	4.51	4.75
Latency (msec）	238	371	236
SR task	post S1	post S2	post S3
N100			
Amplitude (μV)	−0.54	−1.27	−0.53
Latency (msec）	159	145	150
P300			
Amplitude (μV)	1.95	2.52	2.21
Latency (msec）	239	396	231

*SR, self-reflection task; WM, working memory task; S1, warning stimulus; S2, target stimulus; S3, command stimulus*.

### ERpow

#### Spatial Distribution of ERpow

**Figure 4:**
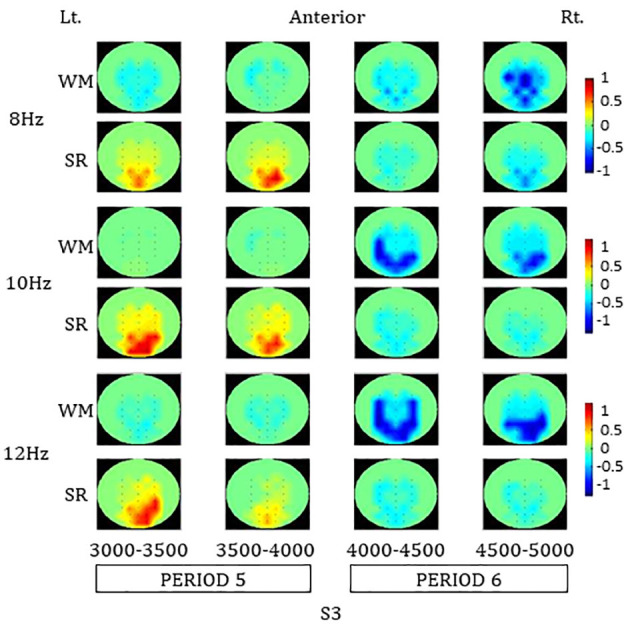
Color maps of spatial distributions of alpha-band ERpow in PERIOD 5 and 6 during the SR and the WM tasks. SR, self-reflection; WM, working memory.

In the color map of spatial distribution (Figure 4) for the SR task, an increase in the ERpow was dominantly observed around parieto-occipital areas during PERIOD 5 at 8, 10, and 12 Hz. A decrease in the ERpow was also observed around parieto-occipital areas during PERIOD 6 in the SR task at 8, 10, and 12 Hz.

The increase in ERpow in each ROI during PERIOD 5 was statistically significant compared to baseline in the earlier part of PERIOD 5 at 8 Hz and in both earlier and later parts of PERIOD 5 at 10 H and 12 Hz, respectively: 8 Hz (PO3: earlier part of PERIOD 5, *F*_(1,30)_ = 5.24, *p = 0.03; later parts of PERIOD 5, *F*_(1,30)_ = 3.74, *p* = 0.06; PO4: earlier part of PERIOD 5, *F*_(1,30)_ = 4.47, *p = 0.04; later part of PERIOD 5, *F*_(1,30)_ = 8.64, *p = 0.01), 10 Hz (PO3: earlier part of PERIOD 5, *F*_(1,30)_ = 8.70, *p = 0.01; later part of PERIOD 5, *F*_(1,30)_ = 15.61, **p < 0.01; PO4: earlier part of PERIOD 5, *F*_(1,30)_ = 14.32, **p < 0.01; later part of PERIOD 5, *F*_(1,30)_ = 19.32, **p < 0.01), and 12 Hz (PO3: earlier part of PERIOD 5, *F*_(1,30)_ = 5.45, *p = 0.03; later part of PERIOD 5, *F*_(1,30)_ = 9.20, *p = 0.01; PO4: earlier part of PERIOD 5, *F*_(1,30)_ = 12.88, **p < 0.01; later part of PERIOD 5, *F*_(1,30)_ = 13.94, **p < 0.01).

In the color map of spatial distribution for the WM task, a decrease in ERpow was observed involving the central and parieto-occipital areas during PERIOD 6 at 8, 10, and 12 Hz. A greater decrease in ERpow in the WM task compared to the SR task was observed in those areas during the PERIOD 6.

#### Time Series of ERpow

**Figure 5:**
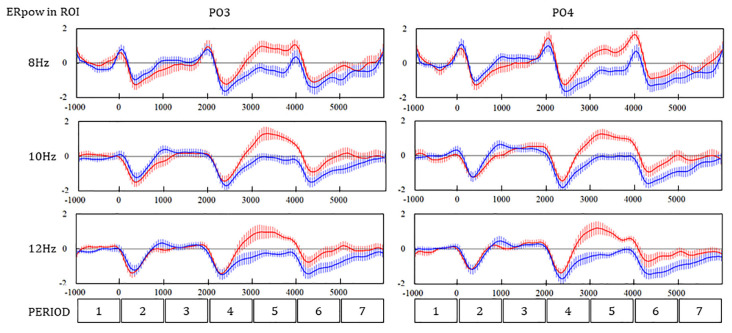
Time series of ERpow at PO3 and PO4 during the SR and WM tasks. Redline, the self-reflection (SR) task; Navy line, the working memory (WM) task.

A great difference in ERpow in PO4 and O1 between the WM and SR tasks was observed during the later part of PERIOD 4 and PERIOD 5. The ERpow of 8 Hz remarkably decreased and recovered at baseline in the WM task and weakly decreased and steeply increased in the SR task (Figure 5). The time series of ERpow of 10 Hz and 12 Hz showed a trend similar to that of 8 Hz. The ERpow of 8 Hz peaked after S3, where the *N* component in ERP peaked, while no ERpow of 10 Hz or 12 Hz peaked.

The three-way ANOVA revealed that the main effect of PERIOD and interaction between the PERIOD and the task were significant in the ERpow of PO3 (*F*_(6,2268)_ = 69.53, *p* < 0.01; **F_(6,2268)_ = 29.74, **p < 0.01) and PO4 (*F*_(6,2268)_ = 70.11, **p < 0.01; *F*_(6,2268)_ = 31.36, **p < 0.01). The interaction between the PERIOD and the frequency was also significant in the ERpow of PO3 (*F*_(12, 2268)_ = 2.07, *p = 0.02) and PO4 (*F*_(12, 2268)_ = 2.65, **p < 0.01). Scheffe’s multiple comparison as a *post hoc* analysis showed a significant difference in the ERpow between the WM and SR tasks in four PERIODs for 8, 10, and 12 Hz in PO4, one PERIOD for 8 Hz, three PERIODs for 10 Hz, and three PERIODs for 12 Hz in PO3 (Table 2).

**Table 2:** The difference in the event-related power change between the working memory task and the self-reflection task in ROIs.

PO3	8 Hz		10 Hz		12 Hz	
	*F*	*p*	*F*	*p*	*F*	*p*
PERIOD 1	3.2	0.08	3.0	0.08	2.0	0.16
PERIOD 2	1.4	0.24	3.0	0.08	0.5	0.48
PERIOD 3	1.8	0.18	1.2	0.27	0.9	0.35
PERIOD 4	1.9	0.17	2.8	0.09	3.1	0.08
PERIOD 5	34.3	< 0.01**	43.3	< 0.01**	34.8	< 0.01**
PERIOD 6	2.6	0.11	11.9	< 0.01**	12.9	< 0.01**
PERIOD 7	2.5	0.12	5.0	0.03*	7.7	0.01*
PO4	8 Hz		10 Hz		12 Hz	
	*F*	*p*	*F*	*P*	*F*	*p*
PERIOD 1	0.4	0.54	0.14	0.71-	1.3	0.25
PERIOD 2	1.4	0.24	2.15	0.14	0.0	0.99
PERIOD 3	1.9	0.17	0.28	0.60	0.3	0.56
PERIOD 4	4.1	0.05*	7.08	0.01*	6.2	0.01*
PERIOD 5	45.1	< 0.01**	55.8	< 0.01**	48.8	< 0.01**
PERIOD 6	4.2	0.04*	12.9	< 0.01**	13.8	< 0.01**
PERIOD 7	3.9	0.05*	6.7	0.01*	7.0	0.01*

**p < 0.01, *p < 0.05 by Sheffe’s multiple comparison.

## Discussion

This is the first study to show the discrepancy in the alpha-band ERpow of 8, 10, and 12 Hz in the occipito-parietal areas (PO3 and PO4) between the WM and SR task for a period between a target stimulus and a command stimulus (PERIOD 4 and 5), where a participant had internal attention switched from external attention according to the SR task and remained with external attention according to the WM task. The results suggest that alpha-band ERpow in the occipital-parietal areas are associated with the direction of attention in response to cognitive stimuli, indicating that the findings of ERpow during the two tasks would potentially aid in the clarification of the pathophysiology of a dysfunctional change in attention in patients with psychiatric disorders such as ADHD.

### ERpow in the Time Domain

After S2 in the SR task, subjects were thought to complete recognition of the presented word until the p300 peaked. After that, subjects were thought to internalize their attention until the S3 presentation, because they were asked to respond if they believed the presented word matched one of their personality traits. On the other hand, after S2 in the WM task, subjects were thought to complete not only recognition of the presented word but also judgment whether S2 was matched with S1 until the p300 peaked. After that, subjects were thought to externalize their attention, because they only had to wait for the S3 presentation. In other words, the opposite direction of attention in the SR and WM is paid during PERIOD 4 and 5. In these periods, alpha-band ERpow was increased in the SR task, was decreased relative to baseline in WM, and showed a significant discrepancy between the SR and WM tasks in the occipito-parietal areas.

In the condition that attention turned towards internal, the areas related to DMN are activated (Buckner et al., 2008; Buckner and Dinicola, 2019), and the amplitude of alpha rhythmic activity in such areas is also increased (Mo et al., 2013). Together with the evidence, the results of the current study suggest that the discrepancies of alpha-band ERpow in a period showing the opposite direction of attention are associated with the DMN activation in the SR task and DMN suppression in the WM task. Increased alpha ERpow during the SR task likely indicates DMN activation, while reduced alpha ERpow during the WM task may be indicative of frontoparietal attentional network activation, as this network is anticorrelated with the DMN.

### ERpow in the Spatial Domain

Concerning the spatial distribution of alpha-band ERpow, the increase of ERpow was dominantly mapped in the occipito-parietal areas relevant to the cortical areas of DMN. Changes in alpha rhythmic activity related to the arousal level were dominant in the occipito-parietal areas (Ota et al., 1996; Cantero et al., 1999). In occipital-parietal areas, the alpha power increased to levels similar to a relaxed state, when a task required isolation from the external world so that the attentional direction was kept internalized (Magosso et al., 2019).

Exact low-resolution brain electromagnetic tomography analysis revealed that when the attentional direction was internal, the origin of the alpha oscillatory activity was assigned to mid-occipital-parietal areas related to DMN (Travis et al., 2010). These findings potentially support our result of spatial distribution for the discrepancy of alpha-band ERpow between SR and WM tasks.

### Behavioral Performance

Results of behavioral data showed that the subjects correctly performed the tasks. If the mouse was randomly clicked in response to “Match” and “No Match” trials during the SR task, the significant discrepancy of the rate of the TOP 3 words selected between the two trials was not demonstrated. We cannot exclude the possibility that the participants may have determined the response without their self-reflection before giving the word at S3. The skipping of self-reflection results in the shortening of the reaction time; however, the mean reaction time of the SR task was significantly longer than that of the WM task. The difference partly supports the premise that the subjects engaged in self-reflection during PERIODS 4 and 5 in the SR task. Although the significant difference in the reaction time between the tasks does not sufficiently exclude the possibility of skipping self-reflection only in the latter part of the experiment, which consists of four blocks, the lack of a statistically significant main effect of BLOCK in the one-way repeated measures ANOVA suggested a lower possibility of skipping self-reflection in the latter part. The subjects were thought to correctly perform the WM task because the accuracy rate of the task was over 95%. The current study used English adjectives for the words presented in the SR and WM tasks. We considered that our preliminary study using Japanese words did not show the clear difference of ERpow between the WM and SR tasks, partly because it was too easy for participants to do the task (Shimode et al., 2019). The difficulty of tasks may be due to the evident difference of ERpow between WM and SR tasks in the present study and the longer reaction time in the tasks in the present study than in the preliminary study. Although the presenting of non-native words for subjects likely contributed to the drawing of sufficient attention, habituation for selecting words possibly occurred in the latter part of each experiment that consisted of four blocks because each experiment required over 100 min in our study. However, if such habituation has occurred, the reaction time should be shortened in the latter part of the experiment. The lack of a statistically significant main effect of BLOCK in the one-way repeated measures ANOVA suggested a lower possibility of such habituation. The results of the ERP analysis also support the evidence that subjects performed the two tasks differently. The N100 after S2 in the SR task demonstrated the maximum peak amplitude, which means that the discrimination process showed the most activation in this phase. The largest peak amplitudes of N100 and the longest peak latency of P300 in the WM task was demonstrated after S2, which means that the largest cognitive resource was consumed after S2.

### Contribution to Clinical Research for Mental Disorders

*A prior* study demonstrated the functional association between the transient decrease in alpha-band ERpow and the suppression of DMN during working memory tasks in patients with ADHD (Lenartowicz et al., 2016). However, to the best of our knowledge, there is no report describing the discrepancy of alpha-band ERpow using the two tasks with different directions of attention, such as the WM and SR tasks. The time series of discrepancy in alpha-band ERpow between SR and WM tasks show the possibility that the discrepancy may be associated with a time series of changes in DMN activation, which is potentially helpful in clarifying the pathophysiology of psychiatric patients who have difficulty in controlling the inhibition and activation of DMN activation, such as ADHD.

### Limitations

This study has several limitations. First, we cannot completely exclude the pseudo-peak of ERpow, resulting in the positive-peak of ERpow that was seen at 8 Hz just after S3, although that was seen in 10 and 12 Hz before S3. The pseudo-peak of ERpow at 8 Hz in this study was removed more efficiently than that in our preliminary study (Shimode et al., 2019) because we used the frequency resolution with 2 Hz bin to employ time-domain analysis using the pre-filtering method in the current study while we used the frequency resolution with 4 Hz bin to employ the Fast Fourier Transformation (FFT). Second, we cannot discuss the functional difference of alpha-band ERpow changes across 8, 10, and 12 Hz frequency bins because there was no clear difference between them after removing the pseudo-peak of alpha-band ERpow. Third, we did not analyze gamma-band oscillatory activities although the event-related synchronization in gamma-band oscillatory activities was associated with functional connectivity between cortical areas during a cognitive task (Tallon-Baudry, 2009). The gamma-band oscillatory activity on human scalp EEG was recorded as amplitudes of a few microvolts that resulted in a low signal-to-noise ratio; thus, many epochs would be required to obtain a reliable time series of gamma-band ERpow. In the current study, as the length of the epoch was designed as 7,000 ms, it was hard to obtain many epochs to analyze the gamma-band because the epoch was over 35%, excluding ratios with artifacts such as blinking or EMG. Fourth, we did event-related power analysis on human scalp EEG but not an advanced spatial-temporal analysis like extracting the neuronal network patterns of task-related-oscillatory activity employing Independent Component Analysis (Bowman et al., 2017). This was because we did not have much EEG data to analyze such methods by an EEG device for clinical usage. We wanted to have some evidence using the EEG device for the clinical assessment of psychiatric patients. However, future studies are required to elucidate the spatial-temporal specificity of the alpha-band ERpow discrepancy between the SR and WM tasks using more advanced methods (Bocharov et al., 2019). Fifth, the effect of habituation in the latter part of the experiment was not excluded completely. In future studies, improving the method of epoch selection to obtain additionally available epochs from each block will shorten the total time of the experiment and will likely contribute to reducing the effect of habituation. Sixth, the participants were young graduates and the results cannot be generalized to the general population.

## Conclusion

The current study showed the discrepancy of alpha-band ERpow of 8, 10, and 12 Hz in occipital-parietal areas between the two cognitive tasks with different directions of attention. The results suggest that alpha-band ERpow in occipital-parietal areas is associated with the direction of attention in response to cognitive stimuli, indicating that the findings of ERpow during the two tasks would potentially aid in the clarification of the pathophysiology of dysfunctional change in attention in patients with psychiatric disorders such as ADHD.

## Data Availability Statement

The original contributions presented in the study are included in the article, further inquiries can be directed to the corresponding author.

## Ethics Statement

The studies involving human participants were reviewed and approved by the Ethics Review Board, Saitama Medical University. The patients/participants provided their written informed consent to participate in this study.

## Author Contributions

TM conceived and designed the experiments. TM and TS performed the experiments, collected the data, and analyzed the EEG data. TM, TO, and KM discussed the results and wrote the article. All authors contributed to the article and approved the submitted version.

## Conflict of Interest

TM has received speaker’s honoraria from Otsuka Pharmaceutical, Janssen Pharma, Eli Lilly, Pfizer, Meiji Seika Pharma, and Shinogi, and research donations of Novartis and Mitsubishi Tanabe Pharma. TS has received speaker’s honoraria from Otsuka Pharmaceutical, Dainippon Sumitomo Pharma, Meiji Seika Pharma, and Janssen Pharma. TO has received honoraria from Meiji Seika Pharma, Eli Lilly, Mitsubishi Tanabe Pharma, Dainippon Sumitomo Pharma, Otsuka Pharmaceutical, Mochida Pharmaceutical, Kyowa Yakuhin Kogyo, Janssen Pharma, Pfizer, MSD, and Eisai. KM has received honoraria from Kyowa Yakuhin Kogyo, Yoshitomiyakuhin, Pfizer, Janssen Pharma, Otsuka Pharmaceutical, Dainippon Sumitomo Pharma, Mochida Pharmaceutical, MSD, and Eisai.

## Abbreviations

SR: self-reflection
WM: working memory
EEG: electroencephalogram
ERpow: event-related power
ADHD: attention deficit hyperactive disorder
DMN: Default Mode Network
ERP: event-related potentials
MMN: amplitude of mismatch negativity.
